# Systemic Lupus Erythematosus Presenting With Febrile Illness and Hepatitis

**DOI:** 10.7759/cureus.109735

**Published:** 2026-05-27

**Authors:** Anna Laskova, Bohdan Syritsa, Nabil Al-Annouf

**Affiliations:** 1 Hospital Medicine, Reading Hospital, Tower Health, West Reading, USA; 2 Pathology and Laboratory Medicine, Reading Hospital, Tower Health, West Reading, USA

**Keywords:** autoimmune hepatitis, elevated liver enzymes, febrile illness, lupus hepatitis, systemic lupus erythematosus

## Abstract

Lupus hepatitis is a rare initial manifestation of systemic lupus erythematosus (SLE) and may present with abnormal liver function tests. Differentiating hepatic dysfunction related to SLE from other causes, particularly autoimmune hepatitis, is important because management and prognosis differ significantly depending on the underlying etiology. Liver biopsy may play a critical role in distinguishing lupus hepatitis from other causes of hepatic injury. We describe a case of a man with newly diagnosed SLE who presented with prolonged febrile illness and elevated transaminases.

## Introduction

Systemic lupus erythematosus (SLE) is an autoimmune disease that affects multiple organ systems and has a broad spectrum of clinical presentations. Involvement of the liver is relatively common, with up to 60% of patients developing abnormal liver function tests during the course of the disease [1]. The etiology of liver dysfunction in SLE is often multifactorial and may include lupus hepatitis, drug-induced liver injury (DILI), infectious causes, nonalcoholic fatty liver disease, or overlap with autoimmune hepatitis (AIH) [2].

Lupus hepatitis is a distinct hepatic complication in which liver abnormalities are attributed directly to SLE after other causes have been excluded. It has a reported prevalence ranging from 3% to 9.3% among patients with SLE. It is characterized by a mild hepatocellular pattern of liver enzyme elevation associated with other features of active SLE and extrahepatic manifestations. Anti-double-stranded DNA antibodies are usually present, whereas anti-smooth muscle antibodies are typically absent or present at low titers [3]. Liver biopsy findings are generally nonspecific and lack the characteristic features of AIH, such as interface hepatitis, plasma cell-predominant infiltrates, and rosette formation [4]. Lupus hepatitis presenting as the initial feature of SLE is poorly characterized in the literature and appears to be rare, although isolated case reports have been described. It is more commonly identified during the course of established disease [5].

Fever, a common early manifestation of SLE, may further complicate the evaluation of patients with elevated transaminases because infectious etiologies must be excluded first.

In patients with SLE, distinguishing lupus hepatitis from other etiologies of hepatic dysfunction may be challenging because clinical manifestations, serologic abnormalities, and histopathologic findings can overlap. In particular, differentiating lupus hepatitis from AIH is important because prognosis and management differ substantially. Furthermore, autoimmune diseases such as SLE may be associated with false-positive infectious serologies, further confounding the diagnostic evaluation of patients with prolonged febrile illness and elevated transaminases.

We present a case of new-onset SLE in a male patient with prolonged fever, constitutional symptoms, and persistent transaminase elevation initially concerning for infectious or primary hepatic disease. Liver biopsy and comprehensive autoimmune evaluation ultimately supported the diagnosis of lupus hepatitis, with rapid clinical improvement following corticosteroid therapy.

## Case presentation

A 42-year-old man with a remote history of treated hepatitis C virus infection presented with polyarthralgia, 30-lb weight loss, and daily fevers for approximately two months. He also reported swelling and tenderness of the hands, sore throat, diaphoresis, and right upper quadrant abdominal pain. He denied skin rashes, oral ulcers, or hair loss.

He had been admitted to the hospital one month earlier for similar symptoms, underwent an unremarkable infectious workup, and was found to have an unexplained elevation of transaminases. A liver biopsy had been performed, with the results still pending at the time of readmission. His only recent medication was ibuprofen. Pertinent social history included hiking in wooded areas.

Vital signs in the emergency department included BP 117/74 mm Hg, pulse 102 bpm, temperature 100.3 °F, and peripheral oxygen saturation (SpO₂) 95% on room air. Physical examination revealed right upper quadrant abdominal tenderness. No skin rashes or synovitis were identified. 

Laboratory tests on admission demonstrated mild anemia, elevated aspartate aminotransferase (AST), elevated alanine aminotransferase (ALT), and elevated inflammatory markers (Table 1). Bilirubin levels were not elevated.

**Table 1:** Laboratory test results on admission. AST: aspartate aminotransferase; ALT: alanine aminotransferase

Test	Value	Reference range and units
Hemoglobin	10.5	14-17.5 g/dL
White blood cells	7.3	4-10.8*10^9^/L
Platelets	366	150-450*10^9^/L
Creatinine	0.69	0.73-1.18 mg/dL
AST	70	<35 IU/L
ALT	86	10-49 IU/L
Alkaline phosphatase	164	46-116 IU/L
Lactic acid	1.6	0.6-1.5 mmol/L
C-reactive protein	8.7	<0.5 mg/dL
Erythrocyte sedimentation rate	58	0-15 mm/hr
Ferritin	480	11-307 ng/ml

Computed tomography (CT) of the chest, abdomen, and pelvis demonstrated trace left pleural effusion (Figure 1A), mild splenomegaly, and mild nonspecific periportal and perihepatic edema (Figure 1B).

**Figure 1:**
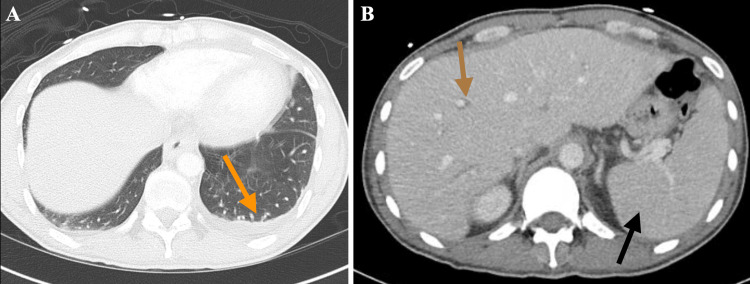
Computed tomography (CT) of the chest, abdomen, and pelvis. A. CT of the chest demonstrating left pleural effusion (orange arrow). B. CT of the abdomen demonstrating mild periportal edema (brown arrow) and mild splenomegaly (black arrow).

The Infectious Diseases service was consulted. Extensive infectious evaluation during both hospitalizations was unrevealing, including testing for streptococcal infection, respiratory viral pathogens, enteric pathogens, parasitic infection, bacteremia, HIV, acute viral hepatitis, and tick-borne illnesses. The only positive infectious test results were Epstein-Barr virus (EBV) IgG (with negative EBV IgM), cytomegalovirus (CMV) IgM, and parvovirus IgM/IgG serologies. Hepatitis C virus and CMV polymerase chain reaction (PCR) were negative. 

The patient was started on doxycycline because of his history of hiking and febrile illness; however, it was later discontinued due to the absence of evidence supporting a tick-borne disease.

The patient continued to experience recurrent high-grade fevers associated with diaphoresis, myalgias, and arthralgias. Daily laboratory studies remained notable for elevated AST and ALT levels, which peaked at 228 and 375 IU/L, respectively.

Multiple autoimmune tests revealed a positive antinuclear antibody titer, elevated anti-double-stranded DNA antibodies, and mildly positive anti-actin (smooth muscle) antibodies (Table 2). 

**Table 2:** Pertinent autoimmune test results.

Test	Value	Reference range and units
Antinuclear antibody titer	1:1280	<1:40
Anti-actin antibodies, IgG	20	<20 U
Anti-double-stranded DNA antibodies	14	<4 IU/ml

Serum IgG was within normal limits. 

Additional autoimmune studies, including complement C3 and C4 levels, rheumatoid factor, anti-SS-A, anti-SS-B, anti-Sm, myeloperoxidase, proteinase 3, antimitochondrial, and soluble liver antigen antibodies, were normal. Antiphospholipid antibody testing was also negative.

Liver biopsy demonstrated nonspecific mild eosinophil-rich portal inflammation without plasma cell infiltrates or interface hepatitis (Figure 2A), along with mild lobular eosinophilic inflammation (Figure 2B). AIH was ruled out. 

**Figure 2:**
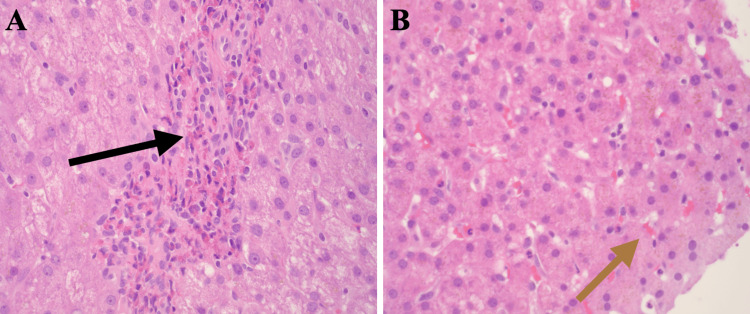
Histopathology. A. Hematoxylin and eosin staining of the liver demonstrating mild portal inflammation composed predominantly of eosinophils and lymphocytes (black arrow). B. Mild lobular eosinophilic inflammation (brown arrow).

Gastroenterology was consulted, and suspected DILI versus lupus hepatitis. After careful consideration, the diagnosis of lupus hepatitis was favored. Rheumatology initiated intravenous methylprednisolone 20 mg every 12 hours for new-onset SLE. Subsequently, both the patient's symptoms and transaminase levels improved (Table 3).

**Table 3:** Transaminase levels trend following steroid initiation. AST: aspartate aminotransferase; ALT: alanine aminotransferase

Steroid treatment day	AST (<35 IU/L)	ALT (10-49 IU/L)
3	228	333
4	137	375
5	32	179
13	28	137
16	12	73

The patient was discharged on a new regimen consisting of a prednisone taper, mycophenolate mofetil 500 mg orally twice daily, and hydroxychloroquine alternating between 200 mg and 400 mg orally daily. He was also referred to an outpatient Rheumatology clinic.

## Discussion

Our patient was admitted to the hospital primarily with constitutional symptoms, including fever, diaphoresis, and weight loss, warranting further evaluation with a broad infectious workup. Given the patient's history of hepatitis C infection, infectious hepatitis was ruled out first. Positive EBV IgG serologies were consistent with prior infection, whereas positive CMV IgM and parvovirus IgM/IgG serologies could not adequately explain recurrent fever lasting more than one month in an immunocompetent patient. These findings prompted further evaluation for autoimmune disease, ultimately leading to the diagnosis of SLE. The isolated positive viral serologies were later attributed to cross-reactivity associated with SLE, a phenomenon previously described in autoimmune diseases [6].

Based on the 2019 American College of Rheumatology and European League Against Rheumatism (ACR/EULAR) classification criteria, the patient met criteria for SLE with a total score of 13 points, including antinuclear antibodies at a titer of 1:1280, anti-double-stranded DNA antibodies, fever, and pleural effusion. The patient had normal complement C3 and C4 levels, which do not exclude SLE flare. Approximately 30-50% of patients with SLE may maintain normal complement levels during active flares [7]. The patient had fever, splenomegaly, and transaminase elevation. However, ferritin levels were not markedly elevated, and cytopenias were absent, making macrophage activation syndrome unlikely based on H-score assessment.

Although the patient demonstrated weakly positive anti-actin (smooth muscle) antibodies, AIH was considered unlikely because serum IgG levels were normal and liver biopsy lacked the characteristic histopathologic features of AIH, including interface hepatitis, plasma cell-predominant infiltrates, and rosette formation. In addition, the degree of transaminase elevation was relatively modest compared with that often observed in active AIH. Distinguishing lupus hepatitis from AIH is crucial because, while lupus hepatitis generally has a favorable prognosis, AIH carries a higher risk of progression to cirrhosis and typically requires long-term immunosuppressive therapy [4]. 

The patient’s liver biopsy demonstrated eosinophil-rich portal inflammation, a nonspecific finding that may also be seen in DILI. Distinguishing DILI from lupus hepatitis may be challenging because of overlapping clinical and histopathologic features. In our patient, liver injury demonstrated a predominantly hepatocellular pattern and occurred in the setting of active disease. Elevated anti-double-stranded DNA antibody levels and serositis with pleural effusion were consistent with an SLE flare. Although the patient had been taking ibuprofen before admission, ibuprofen and other nonsteroidal anti-inflammatory drugs (NSAIDs) were avoided during hospitalization. Despite drug withdrawal, transaminase levels remained elevated and improved only after initiation of SLE-directed therapy. In DILI, liver enzymes generally improve after discontinuation of the offending agent. Although DILI could not be completely excluded, it was considered less likely. Nevertheless, the patient was advised to avoid all nonsteroidal anti-inflammatory drugs (NSAIDs).

Given the clinical, laboratory, and histopathological findings, our patient was diagnosed with new-onset SLE with lupus hepatitis and improved after initiation of systemic steroids. 

## Conclusions

This case highlights lupus hepatitis as a presenting manifestation of new-onset SLE in a male patient with prolonged fever and an extensive negative infectious workup, emphasizing the importance of liver biopsy in distinguishing lupus hepatitis from autoimmune hepatitis. Patients with SLE may develop liver dysfunction from a variety of etiologies, making an accurate diagnosis essential for appropriate management. Liver biopsy may play a critical role in establishing the diagnosis and guiding treatment decisions.
